# Bee and butterfly records indicate diversity losses in western and southern North America, but extensive knowledge gaps remain

**DOI:** 10.1371/journal.pone.0289742

**Published:** 2024-05-15

**Authors:** Sara K. Souther, Manette E. Sandor, Martha Sample, Sara Gabrielson, Clare E. Aslan

**Affiliations:** 1 Center for Adaptable Western Landscapes, Northern Arizona University, Flagstaff, AZ, United States of America; 2 Department of Ecology, Evolution, and Environmental Biology, Columbia University, New York, NY, United States of America; 3 Center for Conservation and Biodiversity, American Museum of Natural History, New York, NY, United States of America; 4 Department of Biology, Northern Arizona University, Flagstaff, AZ, United States of America; National Cheng Kung University, TAIWAN

## Abstract

Pollinator losses threaten ecosystems and food security, diminishing gene flow and reproductive output for ecological communities and impacting ecosystem services broadly. For four focal families of bees and butterflies, we constructed over 1400 ensemble species distribution models over two time periods for North America. Models indicated disproportionally increased richness in eastern North America over time, with decreases in richness over time in the western US and southern Mexico. To further pinpoint geographic areas of vulnerability, we mapped records of potential pollinator species of conservation concern and found high concentrations of detections in the Great Lakes region, US East Coast, and southern Canada. Finally, we estimated asymptotic diversity indices for genera known to include species that visit flowers and may carry pollen for ecoregions across two time periods. Patterns of generic diversity through time mirrored those of species-level analyses, again indicating a decline in pollinators in the western U.S. Increases in generic diversity were observed in cooler and wetter ecoregions. Overall, changes in pollinator diversity appear to reflect changes in climate, though other factors such as land use change may also explain regional shifts. While statistical methods were employed to account for unequal sampling effort across regions and time, improved monitoring efforts with rigorous sampling designs would provide a deeper understanding of pollinator communities and their responses to ongoing environmental change.

## Introduction

Over 85% of global flowering plants, including a third of crop species, are pollinated by animals [1], but declining pollinator populations have been detected in systems worldwide [2–4]. Pollinator declines have been traced to a suite of drivers including biological invasions, disease and parasites, habitat loss, pesticides, monocropping, and climate change [5, 6]. However, reports of declines are typically limited to well-studied taxa, agricultural areas, locations near human communities, and other systems that are easy to access and study [e.g., 7–10]. We lack a comprehensive understanding of which species contribute effectively to pollination or their population dynamics and distributions. Most pollinators are cryptic, and data collection is often limited to circumstances in which observers have access to sufficient training, technologies, sample preservation, and/or record-keeping [11, 12].

Pollinator losses have caused alarm among scientists and policymakers alike because they impact ecological systems at a foundational level [13–15]. Gene flow among plants maximizes their genetic diversity and thus both reproductive output and adaptability in the face of ongoing environmental change [16–18]. Although many plants are capable of self-fertilization or abiotic pollination, most plant species are at least partially dependent on pollinating animals including insects, birds, mammals, and reptiles [1]. Decreased seed output affects the distribution and relative abundance of plants in ecological communities and has the potential to reduce ecosystem resilience following disturbance [19–21]. In terrestrial systems, plants are foundational to all ecosystem services, since they mediate the supporting services (soil formation, photosynthesis, primary production, nutrient cycling, and water cycling) that underlie provisioning, regulating, and cultural services [22].

A broader understanding of geographic trends in pollinator diversity and declines can support conservation and restoration planning as well as public education promoting pollinator-friendly practices and sustainability [23]. Such information can also spur continued ecological research and monitoring of areas with particularly high recorded pollinator diversity, which may act as critical pollinator hotspots, as well as areas that are likely undersampled [24–26]. To facilitate these activities, we set out to assess the current state of knowledge of pollinators of North America (Canada, the United States, and Mexico), examining spatial and temporal trends in presence records of key pollinator families, known and potential pollinator genera, and relatively well-studied pollinator species of concern.

This study consisted of three parts: (1) First, we assessed evidence of spatial patterns over time for four well-known pollinator families: Apidae, Megachilidae, Pieridae, and Papilionidae. We developed species distribution models for North America to assess areas of diversity and change for these families. (2) To further explore spatial patterns of likely pollinator vulnerability with a group for which we have greater taxonomic resolution and greater monitoring focus, we mapped records of species of conservation concern that are known flower visitors and may therefore transfer pollen. (3) We mapped spatial records of a broad set of genera containing flower visitors that have been recorded feeding on pollen, nectar, or flowers and transporting pollen. This provided us with a coarse overview of the state of knowledge of potential pollen-carriers in North America. Our results highlight areas of pollinator vulnerability as well as key knowledge gaps, helping to advance the scientific and management dialogue surrounding pollinator conservation.

## Materials and methods

### Data extraction from GBIF

For all three components of the study, we extracted occurrence records of known and potential pollinators from the Global Biodiversity Information Facility, an international repository for species occurrence data (GBIF.org (2020), GBIF Home Page. Available from: https://www.gbif.org [2 March 2020]). GBIF amalgamates nearly two billion species occurrence records from museum collections to geolocated community science records [27], and as such is an invaluable resource for information on understudied species with little or no representation in the published literature. We queried GBIF at the species level for all North American records of Apidae, Megachilidae, Pieridae, and Papilionidae, as well as for records of all species of conservation concern.

### Part 1: Patterns of spatial and temporal diversity: Apidae, Megachilidae, Pieridae, and Papilionidae

We selected the bee families Apidae and Megachilidae and the butterfly families Pieridae and Papilionidae for spatial analysis because they are relatively well-known and of interest to researchers, with life history traits better described than is the case for many other invertebrate pollinator families. Apidae is the largest family of bees in the world and includes bees important to agriculture (e.g., *Apis mellifera* and *Bombus impatiens*) as well as large numbers of lesser-known bees [28]. A wide diversity of bee life history traits is represented within the Apidae, including both eusociality and solitary habits, ground nesting, stem nesting, and wood nesting, and cleptoparasitism [28], and many Apidae collect pollen on their legs. Megachilidae are mostly above-ground cavity nesters and collect pollen on their abdomens. Because they span a range of resource and foraging requirements, the two families of bees together may act as a proxy for lesser-known groups that require pollen, range in morphology and feeding behavior, and utilize a diversity of nesting resources. The two focal butterfly groups include many charismatic species that are readily identified on the wing (e.g., for Pieridae, the whites and sulfurs; for Papilionidae, the swallowtails), and the two families span wide elevational ranges and wide taxonomic variation in host plant families [29–31], again suggesting that patterns of diversity among these groups may be reliable indicators of broader patterns among less-studied pollinators with overlapping and diverse requirements.

For these four families, we extracted all GBIF records over North America at the species level. We used a records extraction code developed using R version 3.6.2 (R Core Development Team 2016) to extract occurrences of pollinator taxa from GBIF. We queried the GBIF database using the R-package ‘rgbif’ with standard data cleaning protocols (r-package = ‘CoordinateCleaner’) [32]. The code extracted from GBIF the spatial distribution, number of observations, time of each observation, and mode of detection. Although observation number is not a direct measure of abundance since it is biased by detectability, observer effort, and accessibility of sites, low or declining rates of occurrence in the detection database could highlight taxonomic groups that warrant further investigation, and frequency of observation can be used to compare and contrast the state of knowledge of pollinators across regions, time periods, and mode of detection. To summarize outputs by ecoregion and examine patterns of detections over space, we used georeferenced data points from the Ecoregions of North America Level 1 [33, 34], as well as from the North American Land Change Monitoring System Landsat 30m data layers. The use of ecoregions, which are defined as areas containing generally consistent ecosystem type and environmental resources [34], maximizes the potential policy and conservation relevance of our analyses, by linking findings to areas where management needs and strategies may be generally consistent [34].

We used species distribution models (SDMs) to determine the species ranges of 923 species of pollinators within the four families. We analyzed the occurrence records of these species over Canada, the US, and Mexico. We spatially thinned all occurrence records at the species level using a randomization algorithm (*R-package ‘spThin’*) to a minimum of 10 km distance between individual observations. We retained only those species which had 10 or more occurrence records for analysis.

We pulled all 19 bioclimatic variables from WorldClim with a grid resolution of 2.5 minutes [35]. We used WorldClim data because it is available for all of North America, including Mexico, unlike many other sources of climate data, and (2) because for the time period comparison analysis described below we required historical climate data. We chose this low grid resolution because our analyses were continental in extent, and we were looking for broad patterns across multiple species. To accurately model the environment-occurrence relationships of each species, we reduced the geographic extent of the environmental input data to a box encompassing all occurrence points for any given species [36, 37]. We designated boundaries of this box by rounding the maximum latitude and longitude across all observations up to the nearest whole number and rounding the minimum down to the nearest whole number. For each species, we calculated the variance inflation factor between each of the climate variables and every other climate variable. Highly correlated variables were excluded in a stepwise fashion (R package ‘usdm’; R Core Team 2020) [38].

We ran SDMs in an ensemble model framework (R package ‘sdm’ 2016; R Core Team 2020) [39], which accounts for the uncertainty due to different modeling approaches [40–42], consisting of the following modeling approaches: general linear model (‘glm’), general additive model (‘gam’), flexible discriminant analysis (‘fda’), and multivariate adaptive regression spline (‘mars’). Each species model used 10,000 randomly-selected background points, with the exception of nine species that had too small of a geographic distribution to support this many background points, for which the highest number possible, rounded to the nearest 1000, was used [37]. Each species model was subjected to cross validation [43], whereby the data were randomly split into five roughly equal groups, and in each of the five times the model was fit, one group was used as test data and the other four groups were used as training data such that each of the five groups were used as test data for one of the model fits [39]. We ran three iterations of each of five cross-validation sets, resulting in a total of 60 models across the four model methods. We built weighted average ensemble models for each species where the weighted average was calculated based on the cross-validation performance of the model, using the true skill statistic (TSS) as a threshold [39, 44]. We projected the probability of presence for each species back onto North America but limited by the original bounding box to avoid locations of predicted presence where appropriate climatic conditions were identified by the model but no known occurrences exist. We then converted the continuous probability of presence to binary (‘present’ or ‘absent’), using TSS as the threshold [45]. After thresholding, we summed all species either within or across all of Papilionidae, Apidae, Megachilidae, and Pieridae to create species richness maps.

GBIF records the ‘basis of origin’ of each observation when possible; these categories include: *fossil specimens*, *living specimens*, *machine observations* (an observation made by a machine), *material citation* (observation cited in a publication), *preserved specimen* (observation from a herbarium or museum), *human observations* (an observation made by one or more people; almost all digital), and a catch-all of simply *occurrence*, when origin is not established. For all occurrences of known origin, we separate human observation (digital) from all other observations to look at differences through time in collection types and thus separate effects of data collection method from true patterns of occurrence.

To assess change in diversity and distribution over time, we split our data into two time periods: 1939–1979 and 1980–2020. This resulted in two datasets (S3 Table) and had two advantages: (1) the more recent time period contains almost all of the digital records, so is a different type of data; and (2) rapid increases in average temperatures have been recorded in North America since 1980, such that abiotic conditions across the continent are different between the two time periods. Because of this abrupt shift in methodologies and climate aligning to the time periods, changes in richness or distribution of taxa between the time periods may be driven by either altered detection or altered climate context, whereas patterns observed within a given time period are less likely to be artifacts of either of these influences but may instead be due, for example, to other forms of environmental change. From the 1980’s to the present, each decade was hotter than the last [46], giving the 1980–2020 time period a more pronounced signature of climate change that we could compare to a historical period with less climate change impact. (We recognize that climate change may not be the only driver of species’ range shifts within pollinators. While land use change may be the largest driver of pollinator range shifts, historical data for Mexico is not available to the same degree as for the U.S. and Canada. The countries exhibit differences in land use change and policies (for example, DDT was banned in the U.S. and Canada in 1972 [47] with some use continuing in Canada until 1990 [48] and use in Mexico persisted until 2000 [49]) but data availability and such factors co-vary and are difficult to separate.) Because some species were not recorded in one of the two time periods, these species were discarded. We were left with 736 species across the four families (79.7% of the total) for use in our time-period comparisons.

We pulled climate data by decade from WorldClim version 1 on 12/23/2021, beginning with the earliest decade available, the 1960s. Thus, climate data for the pollinator records from 1939–1979 was compiled from 1961–1979. Climate data for the pollinator records from 1980–2020 spanned the period 1980–2018 because 2018 was the last available year of data. Thus, although the two time periods therefore contained equal numbers of years, the lack of availability of climatic data prior to 1960 resulted in the first time period being trained on the latter 18 years of climate data within the period—a factor that makes our analysis more conservative by potentially diminishing the average climatic differences between the time periods. Across all locations within our dataset, the annual mean temperature for 1980–2020 minus that from 1939–1979 ranged from a minimum of 0 to a maximum of 1.6 (mean = 0.8) degrees C. Mean annual precipitation calculated in the same way ranged from -848 to 400 (mean = 7.19) mm. We created monthly averages for minimum temperature, maximum temperature, and precipitation across each of the two time periods, and cropped each of these to North America. We then created BioClim variables for our two time periods (“biovars” function within the R package ‘dismo’; R Core Team 2020) [50].

We ran SDMs in an ensemble model framework as described above. We created one set of ensemble models for the first time period and one for the second time period. To determine how the predicted ranges of species differed between 1939–1979 and 1980–2020, after thresholding we determined the percentage of area predicted for 1980–2020 as compared to 1939–1979 [51, 52]. We additionally calculated the difference between the predicted range, after thresholding, for each species for 1980–2020 and for 1939–1979. This measure of change determines where species are predicted to have expanded into new areas and where species have been lost from an area in 1980–2020 as compared to 1939–1979.

We transformed the thresholded presence-absence species distribution rasters into shapefiles and calculated the distance and direction of movement of the centroid of the species’ range. We determined the centroid of the range and distance moved from the first to the second time period (R package ‘geosphere’; R Core Team 2020) [53]. We used Haversine distance calculations to get distance output in meters.

### Part 2: Patterns of vulnerability: Species of concern

The SDMs described above were compiled across families, but the lack of resolution in much of GBIF makes it difficult to relate patterns to individual species or to examine potential causes of vulnerability. We addressed this limitation in two ways: (a) we mapped occurrence records for North American pollinator species of concern, which have been relatively well-studied due to their known rarity and relevance to management and policy; and (b) we included vertebrate pollinators, for which species-level records are more reliable, in both parts 2 and 3 of this study. We constructed a list of the species of conservation concern across the three countries in order to analyze spatial occurrence of such species and identify regions of vulnerability. We used the International Union for the Conservation of Nature (IUCN) Red List, version 3, to extract conservation status for all nectivorous vertebrates in the database. The IUCN Red List uses expert consensus to assess species worldwide and assign them to threat-level categories including Extinct, Extinct in the Wild, Critically Endangered, Endangered, Vulnerable, Near Threatened, Least Concern, and Data Deficient. Assessments indicate the quantity of population decline or range contraction that has been observed for a particular species or is deemed likely based on current threats to that species. The IUCN Red List provided an initial list of 11 vertebrate pollinators of conservation concern for North America (S2 Table). Although the IUCN Red List is far less developed for invertebrates than for vertebrates, we also downloaded from the Red List webpage the IUCN conservation status of all invertebrates that may interact with flowers for any of the three focal countries. Twenty-five of these invertebrates (37%) were rated as Near Threatened or in worse status on the Red List. We additionally examined national and state/province-level conservation assessments to seek other potential pollinator species of conservation concern. At the national level, these assessments included listings by Canada’s Committee on the Status of Endangered Wildlife in Canada (COSEWIC) (https://www.canada.ca/en/environment-climate-change/services/species-risk-public-registry.html), the US Endangered Species Act (16 U.S.C. 1531–1544) and the Mexican Official Norm NOM-059-SEMARNAT-2010 (*Protección ambiental-Especies nativas de México de flora y fauna silvestres*). At the state or province level, these assessments included Canadian province listings (obtained by examining each provincial government website), US state listings (obtained by examining each state government website as well as, when those websites referred to them in their listings, State Wildlife Action Plans), and Mexico’s *Estrategias Estatales de Biodiversidad* (https://www.biodiversidad.gob.mx/region/EEB/estrategias.html). Once we had used these lists to develop a database of species of conservation concern that are known or likely to feed on pollen, nectar, or flowers, we applied our GBIF query code at the species level to each species of concern in turn to obtain its observation number and frequency, spatial distribution, and ecoregion. We then fed all outputs into our database to summarize occurrence of threatened species spatially and taxonomically. These overviews allowed us to compare species-level patterns with the less-resolved family and genus-level patterns provided in parts 1 and 3 of the study, enabling validation and biological interpretation. Relating patterns of decline, vulnerability, and richness to known distributions of species of concern allow us to consider the implications of such patterns for conservation and monitoring.

There are 1159 species of concern listed in international (59 species), national (35 species), or state/provincial sources (1065 species). Vertebrates of concern include four bats and seven hummingbirds, all ranked as Near Threatened, Vulnerable, Endangered, or Critically Endangered by the IUCN (S2 Table). Among invertebrates, 4.3% of species of concern are Diptera, 3.9% are Coleoptera, 13.4% are Hymenoptera, and 78.4% are Lepidoptera. Here a likely knowledge gap emerges: Lepidoptera are more easily detected and identified in the field than the other insect groups, and it is likely that records of their occurrence and decline are therefore more complete [54].

### Part 3: Diversity assessment of potential pollinator occurrence across North America

To explore geographic and temporal patterns of potential pollinator occurrence, we used existing studies of pollination and lists of pollinators in North America to assemble a full genus list of potential pollen carrying taxa with which to query GBIF using the code above. We deliberately took a broad, umbrella approach to this analysis. Pollination is messy, with most plants producing large numbers of pollen grains and most pollen lost to the environment [55–57]; even plants that exhibit evidence of pollination syndromes and likely have a single or small number of high-efficiency pollinators are often found to receive pollen from other flower-visiting taxa as well [58]. Likewise, some flower visitors transfer extremely small number of pollen grains in an incidental fashion yet contribute measurably to plant reproduction, whereas others (especially bees) transfer thousands of pollen grains directly between plant individuals [59, 60]. To account for the diffuse nature of pollination networks and the possibility that many taxa provide functional redundancy within systems, we included in our search all genera known to contain taxa that have been recorded as carrying pollen [61]; in addition to nectarivores, palynivores, and florivores, such genera also include omnivores and other opportunistic visitors that pick up pollen in the course of their foraging. Knowledge gaps assessed through this process thus indicate areas of undersampling of the community of potential pollinators in the broadest sense.

To assemble this list of genera, we used overviews and databases of pollination, pollinators, and pollen-carrying taxa in North America, including Discover Life (https://www.discoverlife.org/), the Biosystematic Database of World Diptera (https://www.gbif.org/dataset/f49035c5-335a-418f-bafb-24e0ce03cb27), and BugGuide (https://bugguide.net/node/view/15740, and published sources [62–115] (S1 Table). Although taxonomy among invertebrates is often poorly resolved and genera can be frequently redefined, we nevertheless select genus level for this extraction for several key reasons. We aim to capture as much as possible the full diversity of potential flower visitors, yet only a small fraction of invertebrate species beyond the bees and butterflies have been examined for diet or pollen transport, so a species-level analysis would be too restricted in scope. Family, by contrast, is far too coarse to assume similar form and function within a taxonomic group. Many potential pollinators are difficult to identify to species without advanced taxonomic skill and technology, and genus-level records in many cases are therefore likely more reliable than species-level. There is also large variation in nomenclature applied to samples in GBIF, particularly when samples span many decades of collection date, and use of genus instead of species accounts for many synonyms [23]. This component of our study aims to take a broad, umbrella approach to identify key knowledge gaps, which mitigates the imprecision that accompanies the use of genera for invertebrates since our key take-home messages center on where detections are most prevalent and which areas seem under-sampled.

To include vertebrate pollinators in North America, we included the list of known pollen-carriers provided in Aslan et al. [62], which includes 228 vertebrates including icterids, picids, tanagers, hummingbirds, and nectar-feeding bats. Among bird pollinators, major families include Icteridae (23 species), Cardinalidae (9 species), Thraupidae (13 species), Fringillidae (11 species), Parulidae (9 species), and Trochilidae (109 species). Mammalian pollinators include 16 species of nectarivorous bats (Phyllostomidae). For each of these vertebrate species, we used the query code described above to extract from GBIF observation number and frequency, spatial distribution, observation time horizon, and ecoregion for the full list of vertebrates.

To quantify generic diversity of potential pollen-carrying taxa across ecoregions, we calculated incidence-based Hill numbers in R (version 3.6.3.) package *‘iNext’*. Hill numbers follow a doubling property; that is, if you combine two equally large and diverse assemblages that share no taxa, the resulting assemblage will be twice as diverse as the individual assemblages, allowing meaningful comparisons across data types, including taxonomic, phylogenetic and functional diversity data [116, 117]. Generic richness (*q* = 0), exponential Shannon entropy (*q* = 1), and inverse Simpson index (*q* = 2) are united using the parameter *q*, which specifies the sensitivity of the parameter to the relative abundance of genera, with richness (*q = 0*) insensitive to changes in frequency and Hill numbers of order *q* = 2 the most sensitive to the frequency of genera.

Comparing raw diversity data is inadvisable since estimates are sensitive to sampling intensity/sample size and completeness [117]. To account for differences in sampling intensity, we calculated asymptotic diversity estimates based on rarefication-extrapolation curves, extrapolating to twice the number of observations of the reference sample (i.e., the time period with the greater observation N) with confidence intervals calculated via bootstrapping (n = 200) [117]. Within each ecoregion, we treated each observation as an occurrence of a particular genus, generating frequency estimates for each genus within a particular ecoregion (incidence data). Diversity patterns were analyzed at the ecoregion level, since these areas are similar in terms of environmental conditions and stressors, and comparing diversity patterns among them may help generate hypotheses regarding mechanisms driving changes. Moreover, generating diversity estimates based on ecological rather than purely spatial (i.e., pixels) boundaries improves accuracy. Samples were rarefied, or observations randomly removed, in order to calculate richness across the same overall sampling intensity (total number of observations at each ecoregion). To extend sampling curves beyond observed occurrences, diversity metrics were extrapolated using sample completeness, a method that estimates false absences based on the number of rare species within an assemblage [118].

### Data limitations and biases

Our approach can be used to detect changes in records of the same taxa over time as well as to infer knowledge gaps and areas of under-sampling. The taxonomic lists we used for our final, coarse, all-potential-pollinator analysis (genera for invertebrates, species for vertebrates) are limited by the extent of our knowledge of organismal diets and behaviors. We do not have lists of all pollinators for all plants in North America, so there are certainly cryptic, rare, or undetected pollinators or pollinator groups that were not included in the lists of likely pollinators we used and thus in our GBIF extraction process. Likewise, our lists of genera likely included some cryptic or understudied groups that do not act as effective pollinators, since we included full genera that occurred on existing lists and overviews and were described as transporting pollen, even when the species within those genera have not been individually studied. Many invertebrate pollinators are exceedingly difficult to detect, catch, and identify, making even the extensive database of GBIF sparse for many taxonomic groups and biased toward more charismatic or visible taxa. Geographic locations are unevenly sampled across the study region. Locations that are remote, topographically complex, or far from human occupancy rarely receive the sampling intensity of locations that are easily accessible. An absence from a particular ecoregion or habitat, therefore, can be interpreted only as a lack of confirmed presence, rather than a true absence.

## Results

### Part 1: Patterns of spatial and temporal diversity: Apidae, Megachilidae, Pieridae, and Papilionidae

**Fig 1:**
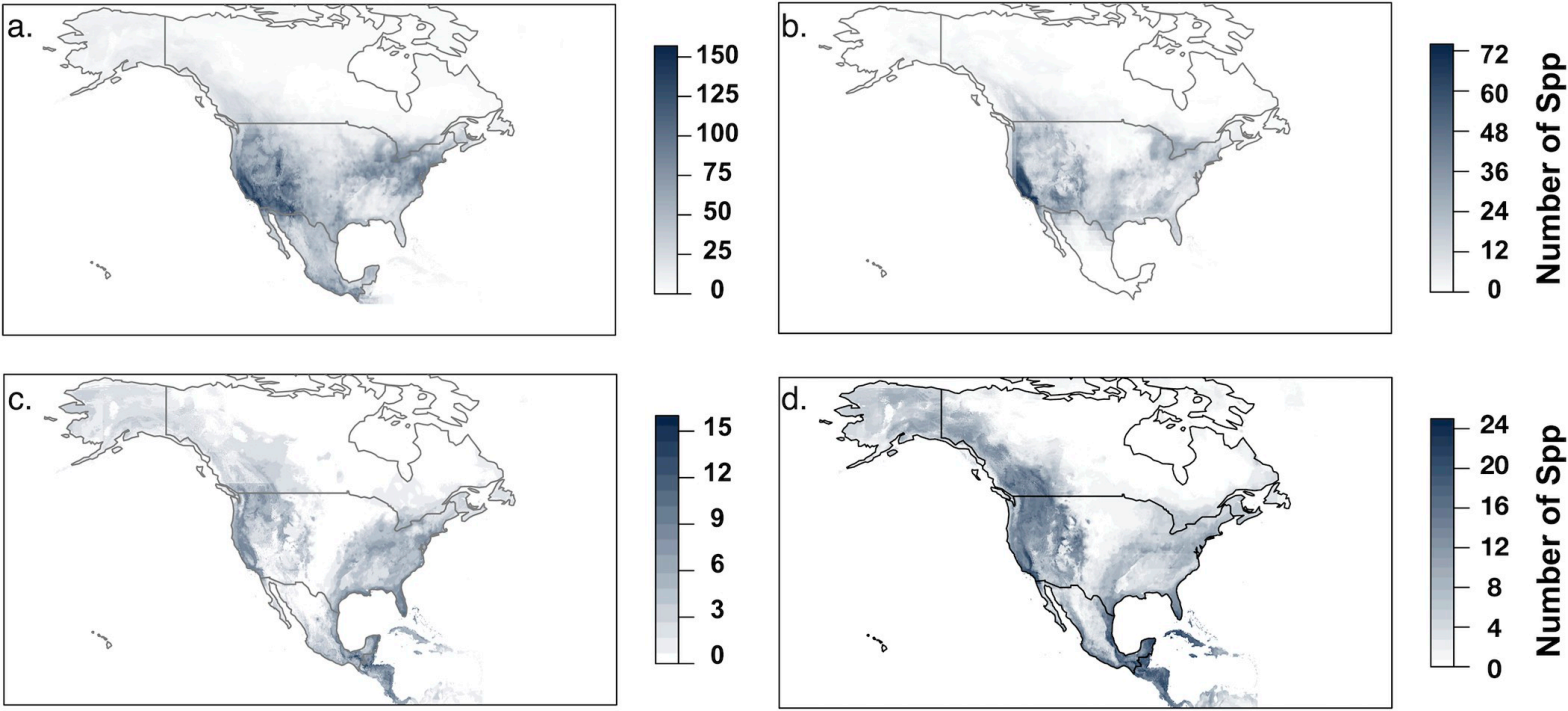
Overlay of species distribution models. Maps include projected distributions of all species from the four families (a) Apidae, (b) Megachilidae, (c) Papilionidae, and (d) Pieridae, depicting locations of highest diversity of species within these families across North America. Maps were created in R version 3.6.2 (R Core Development Team 2016) using thresholded and stacked results of species distribution models for each species, filtered to North American locations, and overlaid.

**Fig 2:**
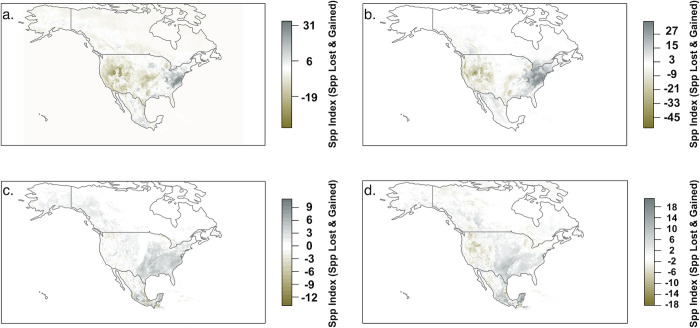
Maps of (a) Apidae, (b) Megachilidae, (c) Papilionidae, and (d) Pieridae species gains and losses within projected species distributions between 1980–2020 and 1939–1979. Thresholded species distribution model outputs from 1939–1979 were subtracted from those from 1980–2020 for all species, resulting in a map of a species distribution with three possible values: -1 (species projected in a given area in 1939–1979 but not 1980–2020), 0 (species projected in a given area in both time periods), and 1 (species projected in a given area in 1980–2020 but not 1939–1979). Each map is the result of the summation of these difference rasters for all species within each family. Areas displayed in gold represent locations where high numbers of species were projected to be lost from the first time period to the second. Dark gray areas represent locations where high numbers of species were projected to be gained from the first time period to the second. Maps were created in R version 3.6.2 (R Core Development Team 2016) using thresholded and stacked results of species distribution models for each species, filtered to North American locations, and overlaid.

Ensemble species distribution models for the four focal charismatic families highlighted different hotspots of high species richness for those focal groups (Fig 1). Highest predicted species richness for the four groups combined occurred along the US West Coast, particularly in California and the Rocky Mountain region, with moderate richness in the Great Lakes and Atlantic seaboard. Time period comparisons among species distribution models for the four focal families indicated that species richness has declined in western North America for all families and, for butterflies, in southern Mexico, and has increased for all families in eastern North America (Fig 2). Centroids of the predicted distributions of analyzed species shifted extensively during the two comparison time periods, with an arithmetic mean percent change for Apidae of 500 (95% CI 490 to 510) km, for Megachilidae of 625 (95% CI 616 to 635) km, for Papilionidae of 487 (95% CI 478 to 497) km, and for Pieridae of 351 (95% CI 342 to 361) km (S1 Fig). Direction of centroid movement varied by species but was most often toward the southeast (S2 Fig). The projected area of species distributions has also expanded substantially between the two time periods, with arithmetic mean percent changes of 137% for Apidae, 202% for Megachilidae, 153% for Papilionidae, and 148% for Pieridae (S3 Fig). Taken together, these findings indicate that in recent decades species’ ranges within these four families have generally shifted southeastward and expanded. The shift and expansion across all four families is likely due to climatic and land-use changes, but other factors, including changes to how the underlying occurrence points were sampled, may be playing a role.

### Part 2: Patterns of vulnerability: Species of concern

**Fig 3:**
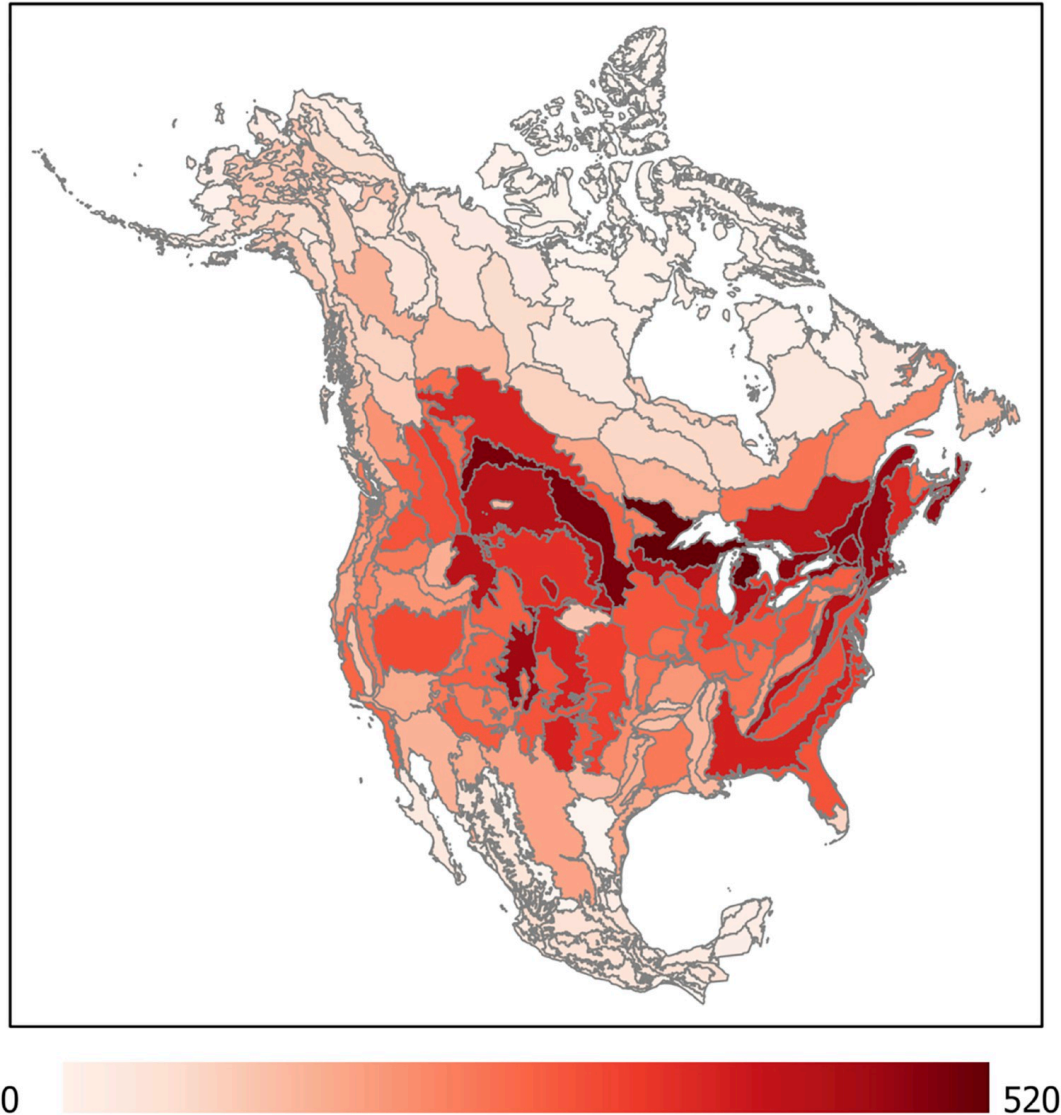
Spatial distribution of occurrence records for likely pollinators that are listed as species of concern, represented by EPA Level III Ecoregions in North America. (Map created in ArcGIS; basemap source: US Environmental Protection Agency, http://edg.epa.gov. Ecoregion reference map available at: https://gaftp.epa.gov/EPADataCommons/ORD/Ecoregions/us/Eco_Level_III_US.pdf).

Records of species of concern were particularly frequent in areas of high human occupancy (the Great Lakes and US East Coast) and across southern Canada (Fig 3); this pattern again tracks areas of high human activity and may also indicate targeted sampling for rare species. Where more sampling is performed, it is more likely that managers and researchers will detect declines over time and thus determine that a species is of concern, and it is also more likely that rare species will be detected. At the same time, areas of high human activity are likely to exhibit high habitat loss and other environmental change, with impact to pollinator populations.

### Part 3: Diversity assessment of potential pollinator occurrence across North America

**Fig 4:**
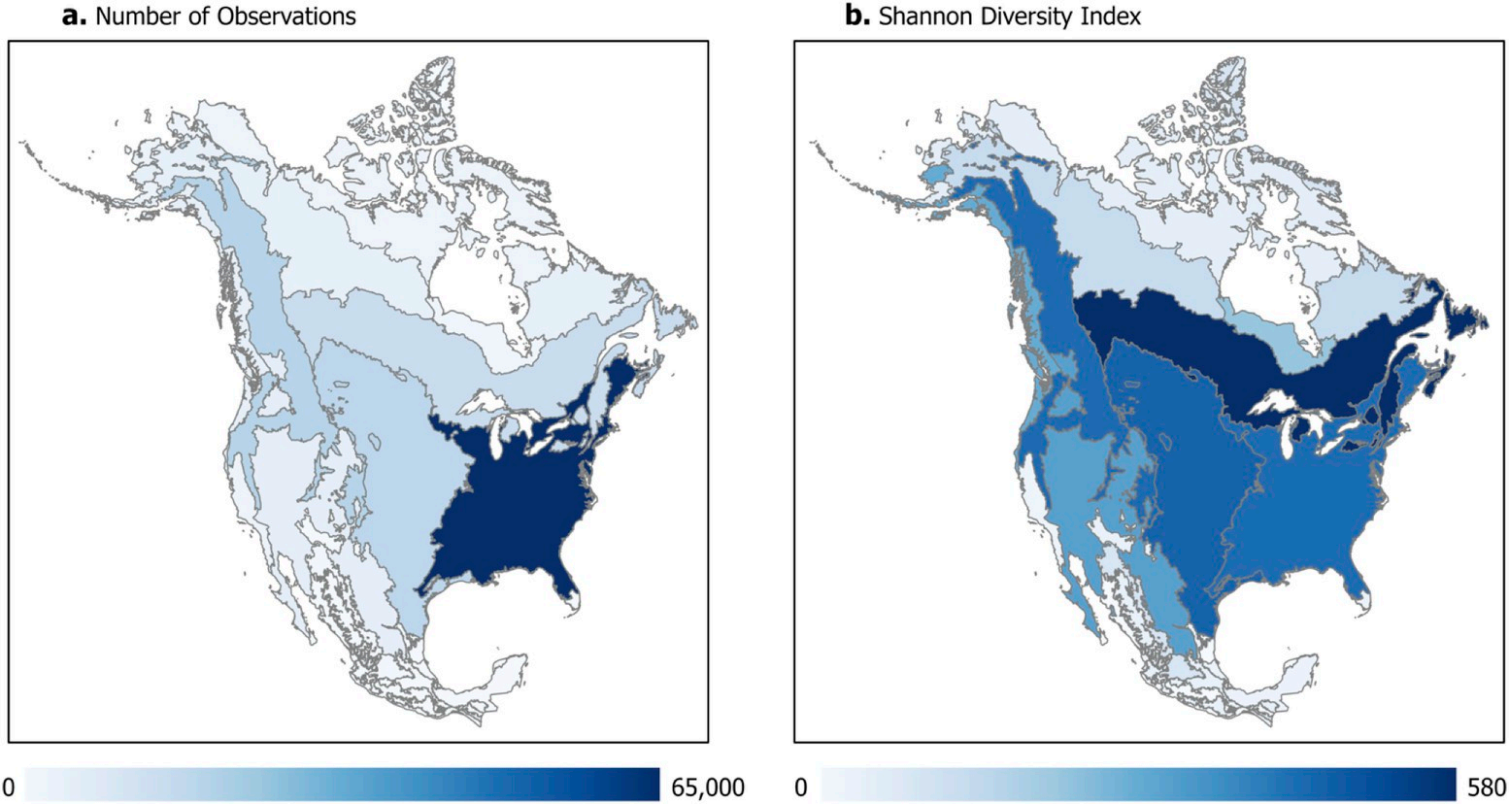
Potential pollinator records across North America. Records are summarized by (a) number of observations in existing GBIF records, and (b) Shannon diversity index within EPA Level 1 Ecoregions in North America. (Map created in ArcGIS; basemap source: US Environmental Protection Agency, http://edg.epa.gov. Ecoregion reference map available at: https://gaftp.epa.gov/EPADataCommons/ORD/Ecoregions/cec_na/NA_LEVEL_I.pdf).

**Fig 5:**
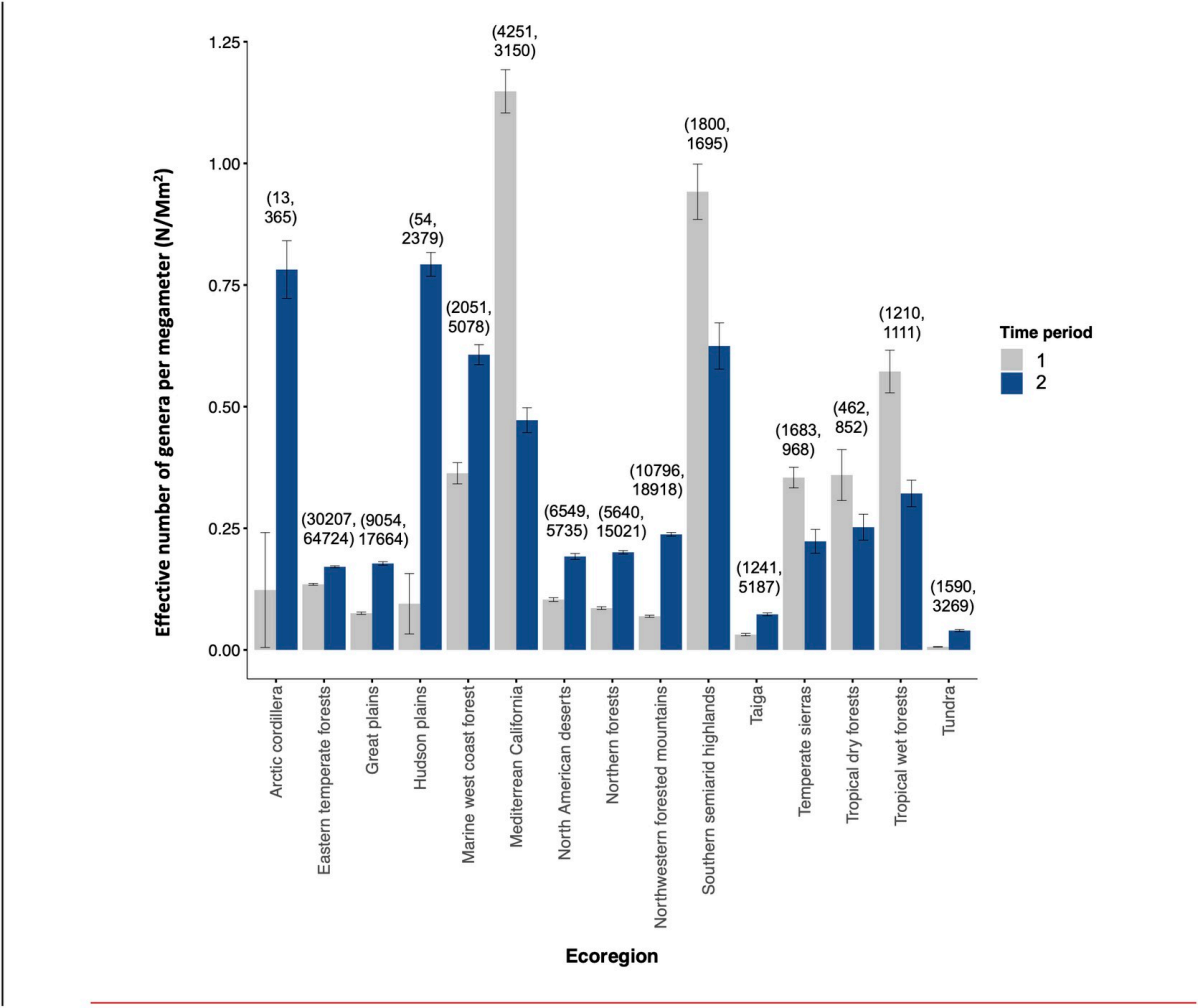
Asymptotic diversity estimates of order q = 1 (equivalent to exponential Shannon entropy) derived from rarefaction-extrapolation curves for each ecoregion (Level 1) relativized by land area (Mm^2^) across two time periods (1939–1979; 1980–2020). Error bars indicate 95% confidence intervals calculated through bootstrapping (n = 200). Observation numbers are presented for each ecoregion above bars, with observations for time period 1 presented first, followed by observation numbers for time period 2. Although with the advent of smartphones and expansion of citizen science efforts detections have increased in most ecoregions, some ecoregions display declining detection between the two time periods.

Analysis aimed at summarizing the distribution of records for all potential pollen carrying taxa included 262,921 observations of 2384 insect genera across the three countries. Across diversity indices, generic richness of potential pollinator occurrence records was highest in the EPA Level I Ecoregions Mediterranean California, Southern semiarid highlands, and Marine west coast forests (Figs 4, 5; S4 Table). When evenness is included in diversity estimates (H = 1, 2), the EPA Level I Ecoregions Hudson Plains and Arctic Cordillera also emerge as contemporary diversity hotspots (S4 Table).

When we examined shifts in generic diversity across all pollinator groups through time, all asymptotic estimates of diversity (H = 0, 1, 2) indicated decreases in diversity over time in Mediterranean California, Tropical Dry and Wet Forests, the Temperate Sierras, and the Southern Semiarid Highlands (EPA Level I Ecoregions; S4 Table). The number of observations differed by several orders of magnitude between time period 1 and 2 for several ecoregions, including the Arctic Cordillera, the Hudson Plains, and the Taiga, which experienced a 27-, 40-, and 3-fold increase in observations, respectively (S4 Table).

## Discussion

The agreement between analyses performed at multiple taxonomic levels across all three parts of our study provides evidence that records of pollinators in at least four key groups (Apidae, Megachilidae, Pieridae, and Papilionidae) have experienced disproportionate increases in the eastern and southeastern US relative to the western US and southern Mexico (Fig 5; S4 Table). Estimates of generic diversity also suggest declining diversity in the western US and tropical regions, while cooler, wetter areas, including the Arctic cordillera, Eastern temperate forests, Hudson plains, Marine west coast forests, Northern forests, Northwestern forested mountains, Taiga, and Tundra, have experienced an increase in pollinator diversity. The one exception to this pattern is the North American deserts ecoregion, where pollinator diversity increased.

Centroid shifting between time periods in SDMs could be due to the influence of sampling on the underlying datasets, the effect of climate change on species’ occurrences, or both. An increase in citizen science and similar data collection efforts could have resulted in oversampling in the East in the second time period, which could then have resulted in the density of underlying presences “pulling” the centroid of the distribution in that direction for many species. Separating digital observations from other observations (i.e., traditional collections) reveals that detections using traditional methods began to decline for all four families in the 1960s although detections using digital observations, as expected, increased sharply after the year 2000 (S4 Fig). Alternatively, a decades-long drought and associated habitat degradation in the West [119–121] could have pushed species’ distributions east. Generally, both methods of assessing shifts in diversity presented here (i.e., SDM analyses and asymptotic diversity analyses) suggest climatic shifts are, at least in part, driving changes in pollinator diversity through time, as pollinators appear to track cooler, wetter conditions. Other factors, such as land use change, may also explain decreases in pollinator abundance observed in areas experiencing high rates of habitat conversion, like the Mediterranean California ecoregion.

Though asymptotic estimates of diversity and SDM approaches account for differences in sampling effort, three ecoregions exhibited order-of-magnitude increases in pollinator observations from time 1 to time 2. Estimates of diversity for these ecoregions, the Arctic Cordillera, the Hudson Plains, and the Taiga, may be inflated due to the sheer magnitude of increase in search effort (S4 Table). Such increased sampling could affect results in areas of high human population and activity (i.e., the eastern temperate forests) but given the high human density across California and parts of Mexico, it is unlikely that disparate sampling effort is the sole cause of unbalanced occurrence records. Instead, areas of reduced detection and high human occupancy may reveal the effects of environmental change on pollinator populations and diversity, particularly where habitat loss has been rapid and climate change-driven drought significant [122, 123]. The enormous number of records available in GBIF allows us to examine changes over time for key groups and to identify areas of particular vulnerability where species of concern are concentrated or richness of well-sampled pollinators has declined [124]. However, knowledge gaps are extensive. An absence from a particular ecoregion or habitat can be interpreted only as a lack of confirmed presence, rather than a true absence. Our use of SDMs reduces this bias but does not eliminate it, since SDMs are based in real occurrence data and thus limited by it in accuracy.

Current occurrence records for both species of concern and for the broad, all-inclusive set of potential pollinators in our final analysis are particularly diverse and abundant in the Great Plains, eastern temperate forests, Great Lakes region, and northern forests. The agreement between these datasets provides evidence that patterns are robust to taxonomic resolution and monitoring effort. Furthermore, this agreement suggests that the patterns of richness and declines found for genera in this study hold implications for species-level conservation and policy. As the number of people interested in and trained in natural history increases, sampling effort also increases, with biases toward locations that are most accessible. We therefore expected that records of most pollinator taxa would have increased over time, with steepest increases in well-sampled regions. Today, an increasing percentage of natural history observations are performed via smartphone, using apps such as iNaturalist and others [125]. Partitioning frequencies in detection using smartphones from detection using museum and herbarium specimens, which have remained constant or declined in recent years, can highlight taxa or geographies in which declines in abundance are in agreement across detection modes and therefore may represent true declines. We developed our species distribution models for four particularly well-known groups in order to address these potential sources of bias—taking advantage of the fact that these groups represent diverse life histories and habitat needs, and patterns within these groups may thus be reflective of less well-studied taxa with overlapping requirements. We also employed methods to reduce error (e.g., spatial thinning) in these SDMs and overlaid the resulting spatial models to assess diversity across the study region.

In spite of data gaps and biases, our results hold implications for policy and management. Species distribution models imply increased presence in the East with potential range expansion for many pollinators in the US, with losses in the West. The western US contains large expanses of forests and rangelands that have been substantially altered over decades of fire suppression, overgrazing, recreation, and resource extraction [126–130]. Anthropogenic disturbances interacting with current and ongoing major drought result in widespread ecosystem degradation, loss of functional groups, and diminished biodiversity [131–135]. In southern Mexico, where SDMs also indicate decreased species richness, expansion of pasture and tree plantations have resulted in losses of native forest since the turn of the century [136]. These known environmental changes may help explain the pollinator range shifts and losses we detected here.

In response to these changes, policymakers and managers can prioritize pollinator habitat restoration. An active current area of research is techniques for and efficacy of such restoration efforts, which range from concentrated gardens of pollinator-friendly plants to broad, landscape-scale seeding with native flowering species [137–144]. Objectives of such restorations are to ensure consistent diversity and abundance of pollinator forage plants over the full growing season as well as availability of cover, protection, and pollinator nesting materials. Although managers are increasingly prioritizing such resources in restoration following disturbance, availability of native seed is limited and can lead to high restoration costs and delays, making pollinator-friendly seed production an additional priority area of need [145–147].

In addition to reductions in pollinators in regions facing broadscale environmental degradation, the current state of knowledge suggests that pollinators remain abundant and diverse in many developed and urban areas. This is encouraging and may reflect the fact that managed urban areas often contain added resources such as landscaping and garden plants, surface water features, and reduced predation [148–150]. Pollinator habitat within urban areas can be promoted by use of flowering plants in public spaces, public education to reduce pesticide application, and replacement of lawns with native plants spanning a diversity of functional groups. However, not all pollinators are equally successful in urban areas [149, 150], highlighting the need for pollinator conservation beyond areas of high human population density.

Our results join an expanding literature aiming to understand current and changing spatial patterns of diversity for potential pollinators, with an eye to identifying knowledge gaps and guiding future research to fill those gaps. Most notably, Chesshire and colleagues [23] recently projected and mapped bee species richness for the contiguous United States, finding that existing records are far from complete and that areas of high human density, particularly coastal regions and national parks, exhibit the highest levels of data completeness. A similar data completeness analysis was performed by Shirey and colleagues [151] for North American butterflies. Results suggested once again that butterflies are best-known in areas of high human population and high citizen science activity, and that under-sampling is particularly pronounced for certain taxonomic groups and for geographic areas with high climate change trajectories [151]. In addition to our primary focus—the time-period comparison we employed to assess shifting patterns of diversity for Apidae, Megachilidae, Pieridae, and Papilionidae—our work here complements these previous efforts in its identification of knowledge gaps by providing a broader lens that encompasses potential pollen-carriers such as flies, beetles, and non-bee Hymenoptera. These studies find broad agreement that systematic sampling across ecoregions and approaches to detect an increasing diversity of taxonomic groups are important to inform changes in invertebrate communities into the future.

Moving forward, improved pollinator sampling to detect declines should include enhanced methods of citizen science engagement, increased sampling of cryptic and elusive taxa, and improved ability to link pollinator diversity and occurrence to climate and vegetation metrics, in order to account for cryptic and more difficult-to-detect pollinator groups. Establishment of regular and systematic pollinator sampling in all Level I ecoregions, for example via monitoring stations or periodic bioblitzes, would help to standardize data collection across geographies and fill in important geographic knowledge gaps. Addressing knowledge gaps is challenging, but continued advancement in quantitative approaches as well as expanded and diversified data collection are key. Pollinators are perhaps the most intersectional biotic group of these times of global change: all human and non-human terrestrial communities rely upon them. Improving our ability to track and understand their declines will be critical to our assessment of food security and ecosystem services in the future.

## Supporting information

S1 Table Families of possible invertebrate pollinators for which GBIF records were extracted, along with example sources used to determine whether genera within each family have been recorded feeding on pollen, nectar, or flowers, and transporting pollen. Target families were identified using these and other sources (see main text) and then online sources including Discover Life, BugGuide, and Biosystematic Database of World Diptera were used to examine genera within the families for dietary and pollen-carrying details.
(DOCX)

S2 Table Vertebrate pollinators ranked as species of concern according to the IUCN Red List. (DOCX)

S3 Table Number of occurrences within each family for the time periods 1940–1979 (T1) and 1980–2020 (T2) that were used in building species distribution models. (DOCX)

S4 Table Asymptotic diversity indices (H = 0; Richness, H = 1; Shannon’s, H = 2; Simpsons) for time periods 1 and 2 across Level 1 ecoregions. Total area (kilometers squared) of each ecoregion and number of observations for each time period are provided. Both diversity indices and observation numbers were also relativized by Ecoregion area and expressed as N *per* Mega meter (1000 Km) squared. For each diversity index, 95% confidence intervals are provided below the value in parentheses.
(DOCX)

S1 Fig The distance that the centroid of projected species’ ranges moved between 1940–1979 and 1980–2020. Bee species are represented by circles (Apidae) and triangles (Megachilidae), and butterfly species are represented by diamonds (Papilionidae) and squares (Pieridae). Distance for all species is shown with the y-axis on a log scale to account for the wide range of values. Arithmetic mean percent change in projected area of the species’ distributions for each family are represented by colored lines, yellow for bees (Apidae (500 km) and Megachilidae (625 km)) and blue for butterflies (Papilionidae (487 km) and Pieridae (351 km)). Changes are likely a consequence of both disproportionately increased detection in some areas of the range and decreased occurrence in other areas. Figure was created in R version 3.6.2 (R package ‘geosphere’ by Hijmans et al. 2021; R Core Team 2020).
(DOCX)

S2 Fig Latitudinal and longitudinal change of the centroid from 1940–1979 to 1980–2020 of the project species’ ranges for the two bee families (displayed in yellow) and two butterfly families (blue). To calculate the change of the centroid of projected species’ ranges, we subtracted the coordinates of the centroid for 1940–1979 from the coordinates of the centroid for 1980–2020. To represent the change in location of the centroid, the blunt ends of the arrows are positioned at (0,0) and the pointed end of each arrow is positioned at the calculated difference in longitude and latitude. Each arrow represents one species within the family. Figure was created in R version 3.6.2 (R package ‘geosphere’ by Hijmans et al. 2021; R Core Team 2020).
(DOCX)

S3 Fig Percent change in the projected area of the species’ distributions of all pollinator species between the time periods 1939–1979 and 1980–2020. Bee species are represented by circles (Apidae) and triangles (Megachilidae), and butterfly species are represented by diamonds (Papilionidae) and squares (Pieridae). Percent change for all species is shown with the y-axis on a log scale to account for the wide range of values. Arithmetic mean percent change in projected area of the species’ distributions for each family are represented by colored lines, yellow for bees (Apidae (137%) and Megachilidae (202%)) and blue for butterflies (Papilionidae (153%) and Pieridae (148%)). A dashed line is shown at 100%, representing no change between the two time periods. Any species with points below this dashed line experienced a decrease in their projected distributions from 1939–1979 to 1920–2020, and any species with points above this dashed line experienced an increase in their projected distributions. The mean percent change for all families was >100%. Figure was created in R version 3.6.2 (R package ‘geosphere’ by Hijmans et al. 2021; R Core Team 2020).
(DOCX)

S4 Fig Observations of genera through time (each line indicates 1 genus). Detections using traditional methods (grey) were variable through time, with declines after 2014, although digital methods of observation generally increased in the 2000s.
(DOCX)
