# Supplementary material for: Bee and butterfly records indicate diversity losses in western and southern North America, but extensive knowledge gaps remain
Source: PLoS One. 2024 May 15;19(5):e0289742. doi: 10.1371/journal.pone.0289742 (PMC11095745; doi:10.1371/journal.pone.0289742)
Supplement: S1 Table — Target families were identified using these and other sources (see main text) and then online sources including Discover Life, BugGuide, and Biosystematic Database of World Diptera were used to examine genera within the families for dietary and pollen-carrying details. (DOCX) [file pone.0289742.s001.docx]

**S1 Table.** Families of possible invertebrate pollinators for which GBIF records were extracted, along with example sources used to determine whether genera within each family have been recorded feeding on pollen, nectar, or flowers, and transporting pollen. Target families were identified using these and other sources (see main text) and then online sources including Discover Life, BugGuide, and Biosystematic Database of World Diptera were used to examine genera within the families for dietary and pollen-carrying details.
