# Supplementary material for: Bee and butterfly records indicate diversity losses in western and southern North America, but extensive knowledge gaps remain
Source: PLoS One. 2024 May 15;19(5):e0289742. doi: 10.1371/journal.pone.0289742 (PMC11095745; doi:10.1371/journal.pone.0289742)
Supplement: S2 Table — (DOCX) [file pone.0289742.s002.docx]

**S2 Table.** Vertebrate pollinators ranked as species of concern according to the IUCN Red List.

| **Class** | **Family** | **Species** | **Threat Status** | **Countries** |
| --- | --- | --- | --- | --- |
| Mammalia | Phyllostomidae | *Choeronycteris mexicana* | Near Threatened | Mex, US |
|  | Phyllostomidae | *Leptonycteris nivalis* | Endangered | Mex, US |
|  | Phyllostomidae | *Leptonycteris yerbabuenae* | Near Threatened | Mex, US |
|  | Phyllostomidae | *Musonycteris harrisoni* | Vulnerable | Mex |
| Aves | Trochilidae | *Cynanthus lawrencei* | Near Threatened | Mex |
|  | Trochilidae | *Doricha eliza* | Near Threatened | Mex |
|  | Trochilidae | *Eupherusa cyanophrys* | Endangered | Mex |
|  | Trochilidae | *Eupherusa poliocerca* | Vulnerable | Mex |
|  | Trochilidae | *Lophornis brachylophus* | Critically Endangered | Mex |
|  | Trochilidae | *Selasphorus rufus* | Near Threatened | Mex, US, Can |
|  | Trochilidae | *Thalurania ridgwayi* | Vulnerable | Mex |
