# Supplementary material for: Bee and butterfly records indicate diversity losses in western and southern North America, but extensive knowledge gaps remain
Source: PLoS One. 2024 May 15;19(5):e0289742. doi: 10.1371/journal.pone.0289742 (PMC11095745; doi:10.1371/journal.pone.0289742)
Supplement: S3 Table — (DOCX) [file pone.0289742.s003.docx]

**S3 Table.** Number of observations for each family in each time period.

| **Family** | **Occurrences T1** | **Occurrences T2** |
| --- | --- | --- |
| Apidae | 27862 | 96311 |
| Megachilidae | 12274 | 16411 |
| Papilionidae | 6727 | 114070 |
| Pieridae | 15094 | 47625 |
| Total | 61957 | 274417 |
