# Supplementary material for: Bee and butterfly records indicate diversity losses in western and southern North America, but extensive knowledge gaps remain
Source: PLoS One. 2024 May 15;19(5):e0289742. doi: 10.1371/journal.pone.0289742 (PMC11095745; doi:10.1371/journal.pone.0289742)
Supplement: S4 Table — Total area (kilometers squared) of each ecoregion and number of observations for each time period are provided. Both diversity indices and observation numbers were also relativized by Ecoregion area and expressed as N per Mega meter (1000 Km) squared. For each diversity index, 95% confidence intervals are provided below the value in parentheses. (DOCX) [file pone.0289742.s004.docx]

**S4 Table**. Asymptotic diversity indices (H=0; Richness, H=1; Shannon’s, H=2; Simpsons) for time periods 1 and 2 across Level 1 Ecoregions. Total area (kilometers squared) of each ecoregion and number of observations for each time period are provided. Both diversity indices and observation numbers were also relativized by Ecoregion area and expressed as N *per* Mega meter (1000 Km) squared. For each diversity index, 95% confidence intervals are provided below the value in parentheses.
