# Supplementary material for: Bee and butterfly records indicate diversity losses in western and southern North America, but extensive knowledge gaps remain
Source: PLoS One. 2024 May 15;19(5):e0289742. doi: 10.1371/journal.pone.0289742 (PMC11095745; doi:10.1371/journal.pone.0289742)
Supplement: S1 Fig — Bee species are represented by circles (Apidae) and triangles (Megachilidae), and butterfly species are represented by diamonds (Papilionidae) and squares (Pieridae). Distance for all species is shown with the y-axis on a log scale to account for the wide range of values. Arithmetic mean percent change in projected area of the species’ distributions for each family are represented by colored lines, yellow for bees (Apidae (500 km) and Megachilidae (625 km)) and blue for butterflies (Papilionidae (487 km) and Pieridae (351 km)). Changes are likely a consequence of both disproportionately increased detection in some areas of the range and decreased occurrence in other areas. Figure was created in R version 3.6.2 (R package ‘geosphere’ by Hijmans et al. 2021; R Core Team 2020). (DOCX) [file pone.0289742.s005.docx]

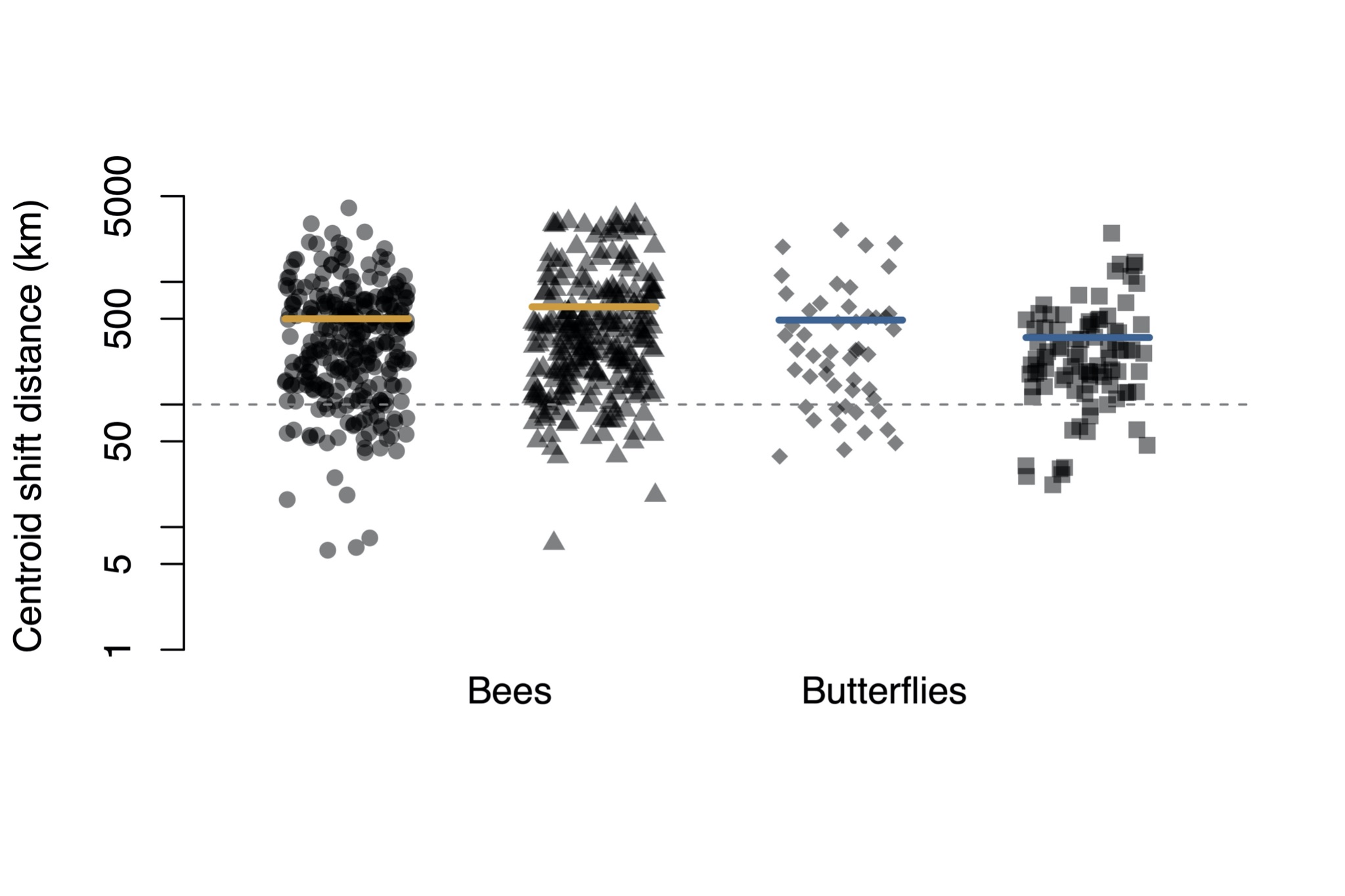


**S1 Fig.** The distance that the centroid of projected species’ ranges moved between 1940-1979 and 1980-2020. Bee species are represented by circles (Apidae) and triangles (Megachilidae), and butterfly species are represented by diamonds (Papilionidae) and squares (Pieridae). Distance for all species is shown with the y-axis on a log scale to account for the wide range of values. Arithmetic mean percent change in projected area of the species’ distributions for each family are represented by colored lines, yellow for bees (Apidae (500 km) and Megachilidae (625 km)) and blue for butterflies (Papilionidae (487 km) and Pieridae (351 km)). Changes are likely a consequence of both disproportionately increased detection in some areas of the range and decreased occurrence in other areas. Figure was created in R version 3.6.2 (R package ‘geosphere’ by Hijmans et al. 2021; R Core Team 2020).
