# Supplementary material for: Bee and butterfly records indicate diversity losses in western and southern North America, but extensive knowledge gaps remain
Source: PLoS One. 2024 May 15;19(5):e0289742. doi: 10.1371/journal.pone.0289742 (PMC11095745; doi:10.1371/journal.pone.0289742)
Supplement: S2 Fig — To calculate the change of the centroid of projected species’ ranges, we subtracted the coordinates of the centroid for 1940–1979 from the coordinates of the centroid for 1980–2020. To represent the change in location of the centroid, the blunt ends of the arrows are positioned at (0,0) and the pointed end of each arrow is positioned at the calculated difference in longitude and latitude. Each arrow represents one species within the family. Figure was created in R version 3.6.2 (R package ‘geosphere’ by Hijmans et al. 2021; R Core Team 2020). (DOCX) [file pone.0289742.s006.docx]

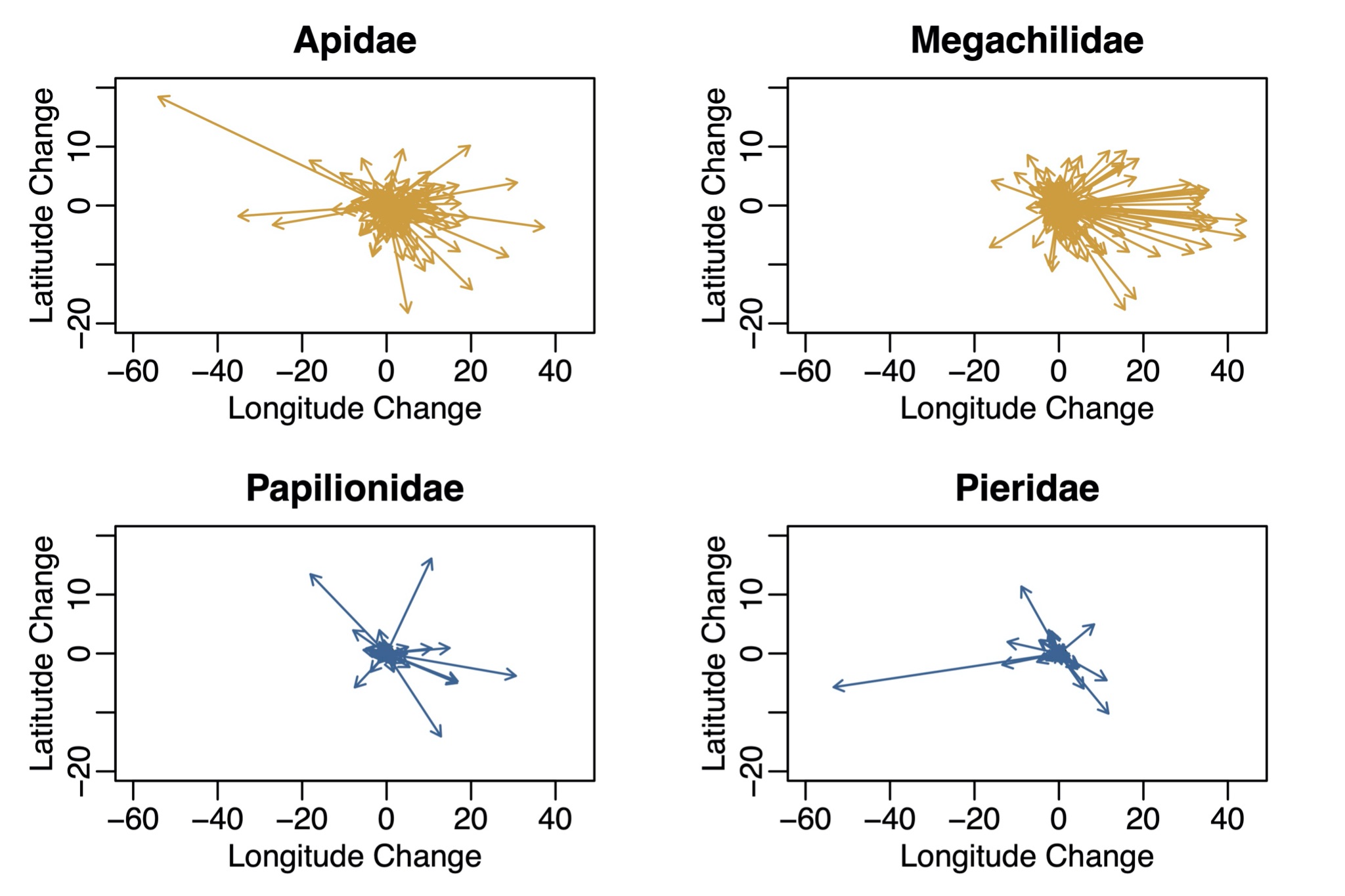


**S2 Fig.** Latitudinal and longitudinal change of the centroid from 1940-1979 to 1980-2020 of the project species’ ranges for the two bee families (displayed in yellow) and two butterfly families (blue). To calculate the change of the centroid of projected species’ ranges, we subtracted the coordinates of the centroid for 1940-1979 from the coordinates of the centroid for 1980-2020. To represent the change in location of the centroid, the blunt ends of the arrows are positioned at (0,0) and the pointed end of each arrow is positioned at the calculated difference in longitude and latitude. Each arrow represents one species within the family. Figure was created in R version 3.6.2 (R package ‘geosphere’ by Hijmans et al. 2021; R Core Team 2020).
