# Supplementary material for: Bee and butterfly records indicate diversity losses in western and southern North America, but extensive knowledge gaps remain
Source: PLoS One. 2024 May 15;19(5):e0289742. doi: 10.1371/journal.pone.0289742 (PMC11095745; doi:10.1371/journal.pone.0289742)
Supplement: S3 Fig — Bee species are represented by circles (Apidae) and triangles (Megachilidae), and butterfly species are represented by diamonds (Papilionidae) and squares (Pieridae). Percent change for all species is shown with the y-axis on a log scale to account for the wide range of values. Arithmetic mean percent change in projected area of the species’ distributions for each family are represented by colored lines, yellow for bees (Apidae (137%) and Megachilidae (202%)) and blue for butterflies (Papilionidae (153%) and Pieridae (148%)). A dashed line is shown at 100%, representing no change between the two time periods. Any species with points below this dashed line experienced a decrease in their projected distributions from 1939–1979 to 1920–2020, and any species with points above this dashed line experienced an increase in their projected distributions. The mean percent change for all families was >100%. Figure was created in R version 3.6.2 (R package ‘geosphere’ by Hijmans et al. 2021; R Core Team 2020). (DOCX) [file pone.0289742.s007.docx]

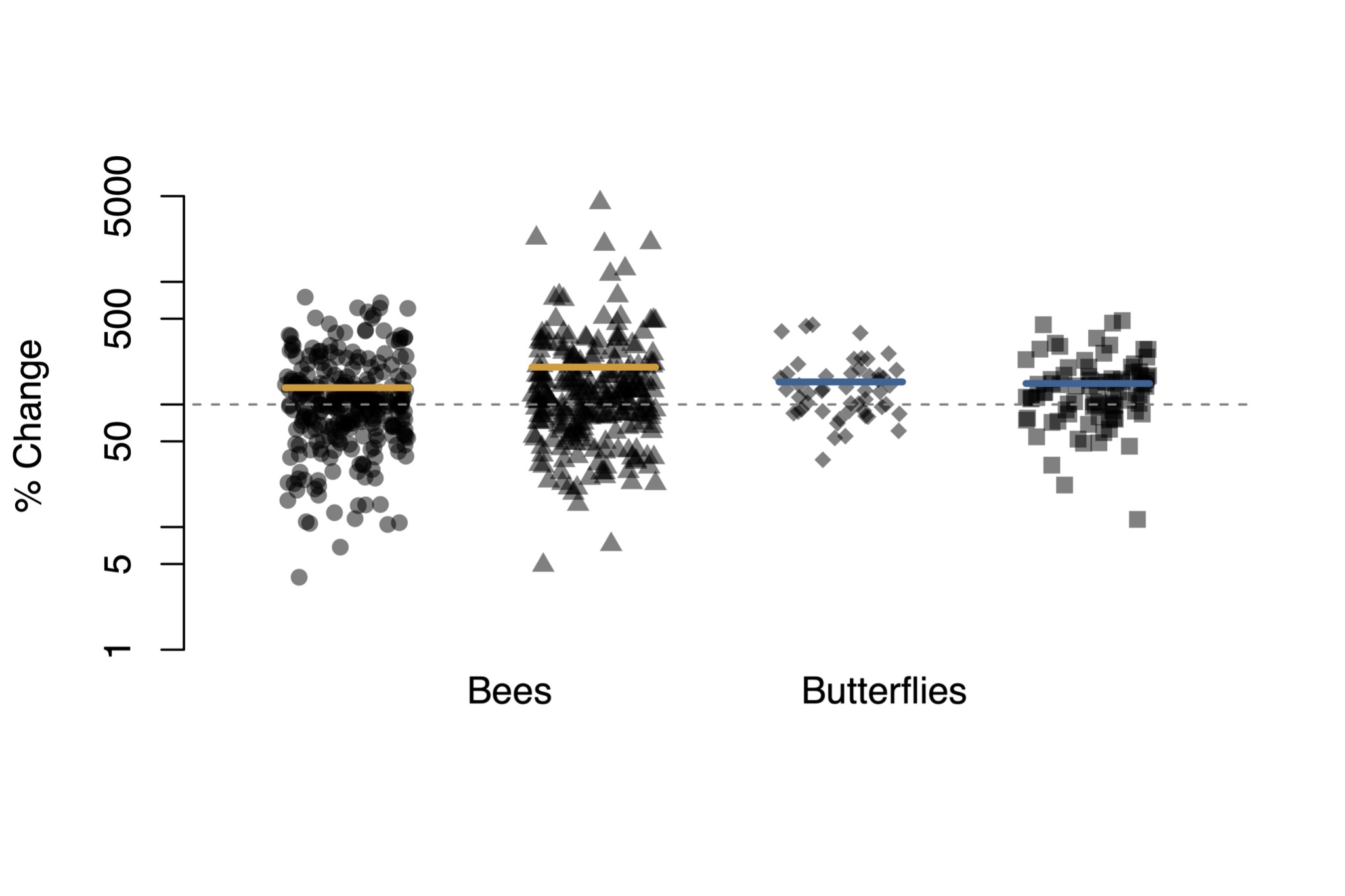
**S3 Fig.** Percent change in the projected area of the species’ distributions of all pollinator species between the time periods 1939-1979 and 1980-2020. Bee species are represented by circles (Apidae) and triangles (Megachilidae), and butterfly species are represented by diamonds (Papilionidae) and squares (Pieridae). Percent change for all species is shown with the y-axis on a log scale to account for the wide range of values. Arithmetic mean percent change in projected area of the species’ distributions for each family are represented by colored lines, yellow for bees (Apidae (137%) and Megachilidae (202%)) and blue for butterflies (Papilionidae (153%) and Pieridae (148%)). A dashed line is shown at 100%, representing no change between the two time periods. Any species with points below this dashed line experienced a decrease in their projected distributions from 1939-1979 to 1920-2020, and any species with points above this dashed line experienced an increase in their projected distributions. The mean percent change for all families was >100%. Figure was created in R version 3.6.2 (R package ‘geosphere’ by Hijmans et al. 2021; R Core Team 2020).
