# Supplementary material for: Bee and butterfly records indicate diversity losses in western and southern North America, but extensive knowledge gaps remain
Source: PLoS One. 2024 May 15;19(5):e0289742. doi: 10.1371/journal.pone.0289742 (PMC11095745; doi:10.1371/journal.pone.0289742)
Supplement: S4 Fig — Detections using traditional methods (grey) were variable through time, with declines after 2014, although digital methods of observation generally increased in the 2000s. (DOCX) [file pone.0289742.s008.docx]

**S4 Fig.** Observations of genera through time (each line indicates 1 genus). Detections using traditional methods (grey) were variable through time, with declines after 2014, although digital methods of observation generally increased in the 2000s.
